# Low-Grade Hepatic Steatosis Is Associated with Long-term Remission of Type 2 Diabetes Independent of Type of Bariatric-Metabolic Surgery

**DOI:** 10.1007/s11695-022-06406-0

**Published:** 2022-12-12

**Authors:** Anne Lautenbach, Marie Wernecke, Oliver Mann, Jonas Wagner, Stefan Wolter, Fabian Stoll, Jens Aberle

**Affiliations:** 1 III Department of Medicine, University Medical Center Hamburg-Eppendorf, 20246 Hamburg, Germany; 2 Department of General, Visceral and Thoracic Surgery, University Medical Center Hamburg-Eppendorf, 20246 Hamburg, Germany

**Keywords:** Liver steatosis, Remission, Type 2 diabetes, Sleeve gastrectomy, Roux-en-Y gastric bypass

## Abstract

**Background:**

Bariatric-metabolic surgery (BS) decreases the grade of steatosis, hepatic inflammation, and fibrosis in patients with severe obesity and non-alcoholic fatty liver disease (NAFLD). Mechanisms include substantial weight loss, but also simultaneous effects on glucose homeostasis. Therefore, we aimed to investigate the association between NAFLD and remission of type 2 diabetes (T2D) up to 8 years following different types of BS.

**Methods:**

In a retrospective cohort study including 107 patients with obesity and T2D at baseline, the association between biopsy-proven NAFLD defined as steatosis in > 5% of hepatocytes at the time of surgery and T2D remission up to 8 years following different surgical procedures was investigated. Univariate regression analysis was used to examine the association between NAFLD and remission of T2D.

**Results:**

Long-term remission of T2D was present in 56% of patients (*n* = 60). The presence of low-grade liver steatosis (grade 1) was associated with remission of T2D. Patients with a liver steatosis score ≥ 2 showed higher HbA1c levels at baseline. There were no significant differences in preoperative presence of lobular inflammation, hepatocyte ballooning, or fibrosis between patients who achieved T2D remission compared with those with no remission. Type of surgery did not affect remission of T2D.

**Conclusion:**

Our results suggest that the presence of low-grade liver steatosis is associated with remission of T2D following sleeve gastrectomy (SG) and Roux-en-Y gastric bypass (RYGB). Therefore, BS should be considered at an early NAFLD stage in patients with T2D.

**Graphical Abstract:**

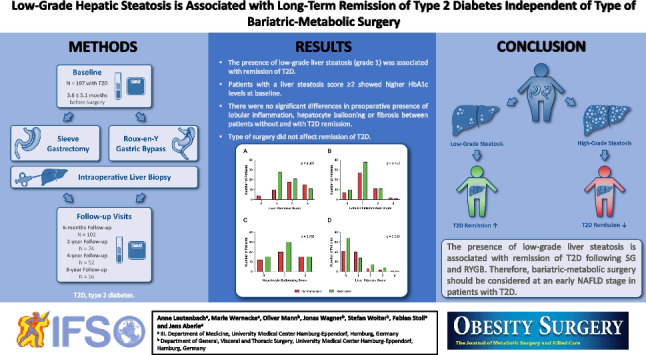

**Supplementary Information:**

The online version contains supplementary material available at 10.1007/s11695-022-06406-0.

## Introduction

The overall prevalence of non-alcoholic fatty liver disease (NAFLD) among patients with type 2 diabetes mellitus (T2D) is 55.5% [1]. Patients with T2D and obesity are at the highest risk of progression from fatty liver to non-alcoholic steatohepatitis (NASH) and cirrhosis [2, 3]. Vice versa, non-alcoholic fatty liver disease (NAFLD) is associated with an approximate twofold higher risk of developing T2D and might precede the development of T2D [2]. The risk of T2D seems to correlate with severity of NAFLD, especially the degree of hepatic steatosis and fibrosis [4]. Current evidence suggests that bariatric-metabolic surgery (BS) decreases the grade of steatosis, hepatic inflammation, and fibrosis in patients with severe obesity [5, 6]. Mechanisms include substantial weight loss, but also simultaneous effects on glucose homeostasis, lipid metabolism, and inflammatory pathways involved in NAFLD pathophysiology [7]. When comparing surgical procedures, Roux-en-Y gastric bypass (RYGB) might be superior to sleeve gastrectomy (SG) in reducing liver steatosis [8, 9]. With regard to diabetes resolution, randomized clinical trials have found no differences in efficacy between RYGB and SG [10–12]. However, according to a large cohort study of 9710 adults with T2D, patients with RYGB seemed to have greater weight loss, a slightly higher T2D remission rate, less T2D relapse, and better long-term glycemic control compared with those with SG comparing 5-year diabetes outcomes [13]. Studies suggest that remission of T2D requires preferential and rapid reduction in liver fat, further supporting the ectopic fat hypothesis for diabetes [14, 15]. Recently, the presence of liver steatosis at the time of surgery has been found to be an independent predictor of long-term diabetes remission following RYGB [16].

Therefore, in the present study, we addressed the following question: Does the presence and severity of NAFLD in patients with obesity and T2D affect long-term diabetes remission following different types of BS?

## Materials and Methods

### Study Population

A total of 367 adult patients (≥ 18 years) who underwent either sleeve gastrectomy (SG) or Roux-en-Y gastric bypass (RYGB) according to the S3 Leitlinie (Guidelines) *Chirurgie der Adipositas* [17] were included in this study. Patients with second step procedures prior were considered as having SG (*n* = 1). Between 2014 and 2022, patients attended our university obesity clinic, which is certified by the European Accreditation Council for Bariatric Surgery as center of excellence for obesity and metabolic surgery. We obtained intraoperative liver biopsies in case of macroscopically suspected liver pathology. Liver biopsies in our cohort have been previously evaluated with a different aim and methodology approved by the ethics committee of the Hamburg Medical Chamber (TV-4889). An independent specialist in gastrointestinal pathology examined the liver biopsies and scored them according to Kleiner et al. [18]. We excluded patients with incomplete records, patients with presence of acute/chronic hepatitis apart from NAFLD, history of acute inflammation (pulmonary, gastrointestinal, urogenital, cutaneous or history of chronic autoinflammatory disease), hyperthyroidism, thyreostatic medication, history of alcohol abuse, and pregnancy (*n* = 72). Of the remaining 295 patients, 188 patients without T2D were excluded (*n* = 107).

All procedures performed in this study involving human participants were in accordance with the ethical standards of the institutional and/or national committee on human research and with the 1964 World Medical Association Declaration of Helsinki and its later amendments or comparable ethical standards. Retrospective data collection and anonymized analysis was conducted in accordance with local government law (HmbKHG. §12) without the requirement for informed consent.

### Study Design

Follow-up data were retrospectively collected from 107 patients at baseline. To provide reasonable comparability between the cases, the available data were allocated 4 “visits” by time in relation to the procedure. In addition to baseline data − 3.6 ± 5.1 (mean ± SD) months before surgery, data from visit 1 (*n* = 100) were analyzed 6.0 ± 2.0 (mean ± SD) months after surgery, data from visit 2 (*n* = 74) 24.0 ± 4.9 (mean ± SD) months after surgery, data from visit 3 (*n* = 52) 47.4 ± 5.9 (mean ± SD) months, and data from visit 4 (*n* = 16) 95.4 ± 7.4 (mean ± SD) months post BS.

### Variables

Data on height (cm), weight (kg), body mass index (BMI; kg/m^2^), sex, age, type of surgery, hemoglobin (g/dl), platelets (10^3^/µl), CRP (mg/l), aspartate aminotransferase (AST; U/l), alanine aminotransferase (ALT; U/l), gamma-glutamyl transpeptidase (GGT; U/l), triglycerides (mg/dl), high-density lipoprotein cholesterol (HDL; mg/dl), low-density lipoprotein cholesterol (LDL; mg/dl) and HbA1c (%) were analyzed at baseline and during follow-up. Excess weight loss (EWL) in % was calculated by dividing the difference between initial BMI and final BMI by the difference between initial BMI and a target BMI of 25 kg/m^2^. Percent of total weight loss (TWL[%]) was calculated by dividing the difference between initial weight and postoperative weight by initial weight multiplied by 100 [19]. Insulin use, use of oral antidiabetic agents and glucagon-like peptide-1 receptor agonists (GLP-1 RA), was assessed at baseline and during follow-up visits. Patients with HbA1c ≥ 7% were defined as having poor glycemic control. Remission of T2D was defined as a return of HbA1c to < 6.5% following BS, that persisted for at least 3 months in the absence of usual glucose-lowering pharmacotherapy [20]. Liver histology was evaluated according to Kleiner et al. [18]. Steatosis was graded on a scale from 0 to 3 with grade 0 defined as < 5% liver fat, grade 1 as 5–33% liver fat, grade 2 as > 33–66%, and grade 3 as > 66%. Lobular inflammation was assessed according to the presence of inflammatory foci on a scale from 0 to 3: no foci (grade 0), < 2 foci per 200 × field (grade 1), 2–4 foci per 200 × field (grade 2), > 4 foci per 200 × field (grade 3). Liver cell injury was recorded as “hepatocyte ballooning” on a scale from 0 to 2: none (grade 0), few balloon cells (grade 1) and many cells/prominent ballooning (grade 2). Fibrosis stage (0–4) was defined as follows: none (F0), perisinusoidal or periportal (F1), mild, zone 3, perisinusoidal (F1A), moderate, zone 3, perisinusoidal (F1B), portal/periportal (F1C), perisinusoidal and portal/periportal (F2), bridging fibrosis (F3), and cirrhosis (F4). The NAFLD activity score (NAS) score was calculated as the sum of scores for steatosis, lobular inflammation and ballooning, ranging from 0 to 8 [18]. A NAS of ≥ 5 corresponded to the diagnosis of NASH, scores of < 3 to “no NASH” [18]. Noninvasive assessment to predict steatosis and fibrosis included the Heaptic-Steatosis Index (HSI) and the Fibrosis-4 index (FIB-4) [21, 22].

### Statistical Methods

Standard descriptive statistics were used for all study end points. Distributions of continuous variables were described with mean and standard deviation (SD). Categorical variables were described with absolute and relative frequencies. Continuous variables at baseline were compared with Student’s *t*-test or Mann–Whitney test. Normality of continuous variables and equality of variances was tested using Shapiro–Wilk test and Levene’s test. Categorical variables were compared using chi-square statistics. Continues data between multiple visits were compared using one-way ANOVA with Dunnett’s test for multiple comparisons. Continuous data between different subgroups over the follow-up period was compared using two-way ANOVA with Šidáks test for multiple comparisons. Multiple logistic regression analysis was conducted to identify significant factors of long-term T2D remission. Odds ratio (OR) with 95% confidence intervals (CI) and *p*-values were calculated. P-values below 0.05 were considered statistically significant.

## Results

### Baseline Characteristics of Patients with T2D

Baseline characteristics are presented in Table 1 (*n* = 107). Mean age was 49.2 ± 11.3 years, 60.7% of patients were female. 69.2% of patients underwent SG, 30.8% underwent RYGB as initial weight loss procedure. Mean HbA1c prior to surgery was 7.6 ± 1.7%. 40.2% of patients were treated with insulin and 55.1% were treated with any oral antidiabetic medication other than insulin. 10.3% (*n* = 11) of patients were treated with GLP-1 RA. Sixty patients (57.0%) showed poor glycemic control.

**Table 1:** Baseline characteristics of patients with T2D

Parameter	Total
	*n* = 107
Sex	
Female	65 (60.7)
Male	42 (39.3)
Age [years]	49.2 ± 11.3
BMI [kg/m^2^]	50.4 ± 9.1
HbA1c [%]	7.6 ± 1.7
Optimal weight loss	
Yes	49 (45.8)
No	58 (54.2)
Preoperative insulin use	
No	64 (59.8)
Yes	43 (40.2)
Preoperative oral antidiabetic agents	
No	48 (44.6)
Yes	59 (55.1)
Preoperative GLP-1RA use	
No	96 (89.7)
Yes	11 (10.3)
Liver steatosis score	
0	4 (3.7)
1	38 (35.5)
2	39 (36.4)
3	26 (24.3)
Liver inflammation score	
0	17 (15.9)
1	65 (60.7)
2	22 (20.6)
3	3 (2.8)
Hepatocyte ballooning score	
0	27 (25.2)
1	50 (46.7)
2	30 (28.0)
Liver fibrosis score	
0	55 (51.4)
1	34 (31.8)
2	10 (9.3)
3	6 (5.6)
4	2 (1.9)
Type of surgery	
SG	74 (69.2)
RYGB	33 (30.8)
Glycemic control	
HbA1c ≥ 7%	61 (57.0)
HbA1c < 7%	46 (43.0)
HSI	64.6 ± 9.8
FIB-4	0.9 ± 0.7

Data are reported as mean ± SD. *N (%)* number of individuals, *T2D* type 2 diabetes, *BMI* Body Mass Index, *GLP-1 RA* glucagon-like peptide-1 receptor agonist, *SG* sleeve gastrectomy, *RYGB* Roux-en-Y gastric bypass, *HSI* hepatic steatosis index, *FIB-4* Fibrosis-4 Index

In total, liver steatosis (grade 1–3) was present in 103 patients (96.2%). Histological diagnosis of NASH defined by a NAS Score ≥ 5 was present in 43 patients (40.1%; data not shown).

84.1% (*n* = 90) of patients showed lobular inflammation (grade 1–3), 74.7% (*n* = 80) of patients showed hepatocyte ballooning (grade 1–2). Histological diagnosis of fibrosis (grade 1–4) was present in 48.6% of patients (*n* = 52), but only 8 patients (= 7.5%) had a score of F3 or F4 corresponding to significant fibrosis [23]. Accordingly, only 2 patients had a FIB-4 score of > 3.25 strongly suggestive of advanced fibrosis [19]. Mean Hepatic-Steatosis Index (HSI) was 64.6 ± 9.8 indicating a high likelihood of NAFLD [21].

### Baseline Histological Characteristics of Patients without and with T2D Remission

**Fig. 1:**
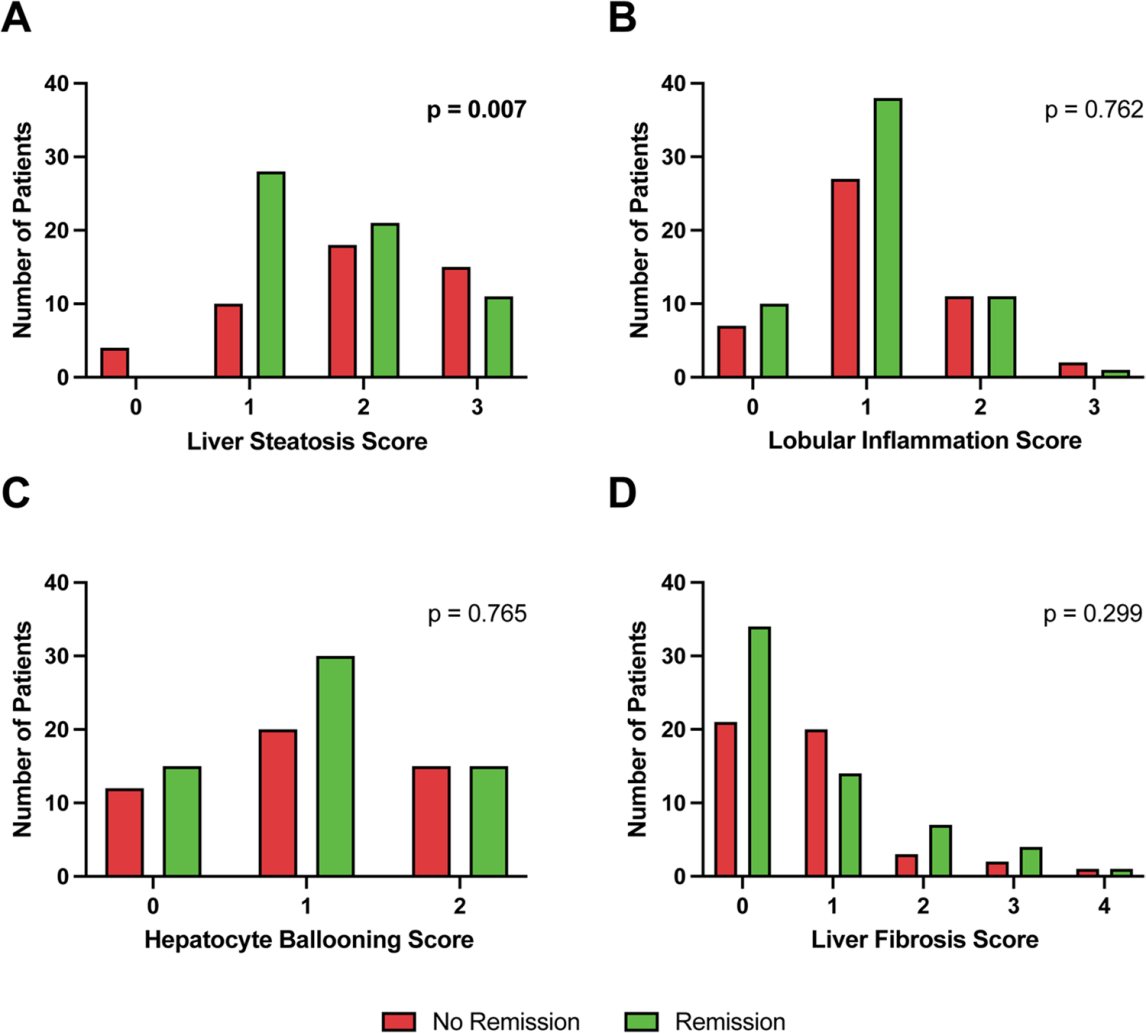
Baseline histological characteristics of patients without and with T2D remission. Liver steatosis score grade 0–3 (**A**), lobular inflammation score grade 0–3 (**B**), hepatocyte ballooning score grade 0–2 (**C**), and liver fibrosis grade 0–4 (**D**) at baseline

Liver steatosis scores at baseline were different between patients without and with T2D remission (*p* = 0.007). Patients with T2D remission showed a higher percentage of low-grade steatosis (score 1) compared with patients without T2D remission. In those patients without T2D remission, steatosis grade 2 was most common. Interestingly, only 4 patients without hepatic steatosis showed no T2D remission. There were no significant differences in preoperative presence of lobular inflammation, hepatocyte ballooning or fibrosis between patients who achieved T2D remission or not post BS (Fig. 1).

### Baseline and Long-term Follow-up Data of Patients Without and with T2D remission

At baseline, patients with T2D remission (*n* = 60) showed a lower mean HbA1c (6.6 ± 1.2 vs. 8.7 ± 1.4%; *p* < 0.001) and lower triglyceride levels (211.0 ± 104.2 vs. 275.9 ± 173.7 mg/dl; *p* < 0.05) than those without T2D remission (*n* = 47). There were no significant differences in liver enzymes, HSI and FIB-4.

**Fig. 2:**
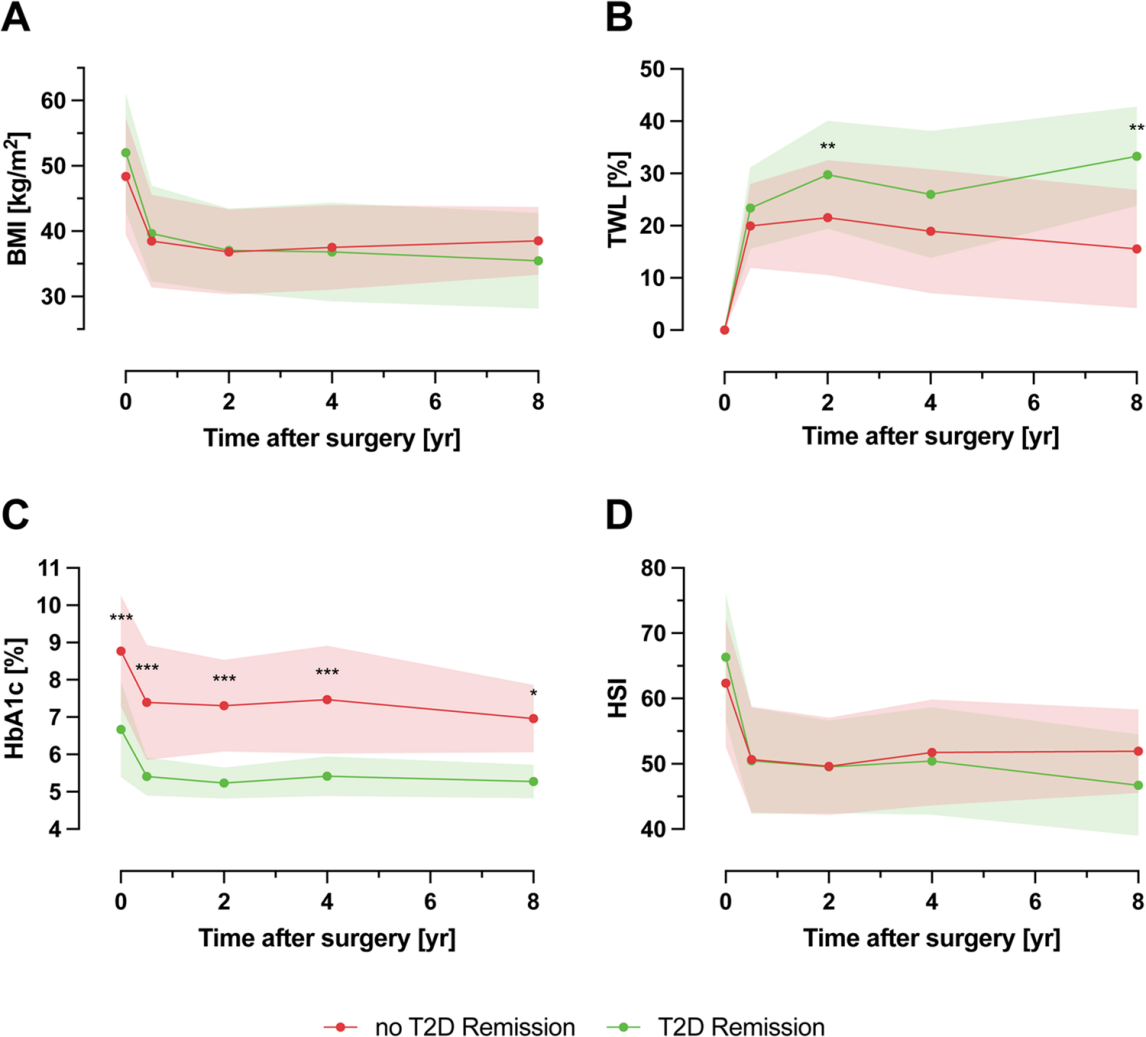
Comparison between patients without and with T2D remission. BMI (**A**), TWL (**B**), HbA1c (**C**), and HSI (**D**) during long-term follow-up. BMI, Body Mass Index; T2D, type 2 diabetes; TWL (%), total weight loss; HSI, hepatic steatosis index; yr, years; *p < 0.005

Mean postoperative follow-up was 95.4 ± 7.4 months. At long-term follow-up, 50% (*n* = 8) of patients achieved remission of T2D. In patients with T2D remission, EWL% (66.1 ± 4.8 vs. 31.7 ± 25.9; *p* < 0.05) and TWL% (33.3 ± 9.5 vs. 15.5 ± 11.3; *p* < 0.005) were significantly higher compared to patients without T2D remission. There were no significant differences in liver enzymes, HSI and FIB-4. In both subgroups, mean HSI was ≥ 36, indicating that NAFLD diagnosis was highly likely. There was a significant decline in HSI in patients without T2D remission (*p* = 0.006) and with T2D remission (*p* < 0.001) (Fig. 2, Table S1).

### T2D Remission at Long-term Follow-up Depending on Liver Steatosis Score

Since T2D remission rates were highest in patients with low-grade liver steatosis (grade 1), we compared patients with liver steatosis scores < 2 and ≥ 2 at baseline. HbA1c, triglycerides, AST, ALT, and GGT were significantly higher in patients with liver steatosis score ≥ 2 at baseline. Long-term, no significant differences were found in weight loss, metabolic and hepatic outcomes (Table 2, Figure S1).

**Table 2:** Comparison between patients with liver steatosis score < 2 (top) and ≥ 2 (bottom)

Parameter	Baseline	*p*	6 M-FU	*p*	24 M-FU	*p*	48 M-FU	*p*	96 M-FU	*p*
Liver steatosis score < 2 [*n*]	42		40		30		20		9	
Liver steatosis score ≥ 2 [*n*]	65		60		44		32		7	
BMI [kg/m^2^]	50.0 ± 8.9		38.3 ± 6.7		35.1 ± 6.8		36.0 ± 7.5		34.3 ± 6.7	
	50.9 ± 9.3	0.985	39.7 ± 7.5	0.918	38.3 ± 5.8	0.345	37.9 ± 6.7	0.930	40.1 ± 4.1	0.583
EWL [%]			46.6 ± 17.9		59.2 ± 29.0		52.0 ± 38.7		52.8 ± 40.7	
			44.5 ± 15.6	0.988	49.1 ± 20.9	0.283	45.3 ± 25.5	0.814	40.2 ± 15.0	0.777
TWL [%]			22.4 ± 9.3		28.2 ± 11.8		25.1 ± 14.7		26.0 ± 18.0	
			21.5 ± 7.3	0.987	24.9 ± 10.9	0.598	21.1 ± 10.9	0.606	20.3 ± 7.9	0.772
HbA1c [%]	7.0 ± 1.4		5.8 ± 0.6		5.8 ± 1.1		5.9 ± 1.2		5.9 ± 1.3	
	8.0 ± 1.8	0.002	6.6 ± 1.7	0.029	6.3 ± 1.4	0.673	6.7 ± 1.6	0.214	6.4 ± 0.9	0.980
Hb [g/dl]	13.9 ± 1.4		13.6 ± 1.2		13.4 ± 1.5		13.8 ± 1.1		13.4 ± 1.0	
	14.0 ± 1.6	> 0.99	13.6 ± 1.4	> 0.99	13.3 ± 1.4	> 0.99	13.5 ± 1.6	0.967	12.8 ± 2.3	0.942
Leukocytes [10^9^/l]	9.9 ± 2.5		8.9 ± 2.5		7.8 ± 2.1		8.4 ± 2.5		7.7 ± 2.3	
	9.5 ± 2.6	0.906	8.9 ± 2.2	> 0.99	8.1 ± 2.6	0.998	8.3 ± 2.0	> 0.99	7.5 ± 1.4	> 0.99
Platelet count [10^9^/l]	296.2 ± 85.5		286.3 ± 83.9		272.1 ± 94.2		294.5 ± 75.5		284.1 ± 59.6	
	290.6 ± 70.5	0.998	285.4 ± 70.0	> 0.99	279.3 ± 71.7	0.997	275.6 ± 63.9	0.908	239.1 ± 35.5	0.743
Triglycerides [mg/dl]	184.3 ± 89.8		154.5 ± 75.3		147.1 ± 83.0		138.6 ± 50.7		128.3 ± 50.5	
	275.2 ± 157.8	< 0.001	214.5 ± 95.8	0.072	222.7 ± 122.9	0.043	280.5 ± 184.4	< 0.001	273.6 ± 196.1	0.081
HDL [mg/dl]	44.0 ± 11.3		44.9 ± 10.8		56.9 ± 12.2		67.4 ± 13.7		69.0 ± 10.5	
	38.6 ± 14.4	0.191	41.9 ± 11.3	0.790	51.1 ± 16.2	0.286	52.0 ± 15.6	< 0.001	52.6 ± 15.0	0.083
LDL [mg/dl]	103.6 ± 34.8		101.5 ± 37.8		86.2 ± 35.1		81.5 ± 28.8		107.0 ± 36.6	
	97.4 ± 43.7	0.947	104.6 ± 40.3	0.998	106.3 ± 41.6	0.186	101.6 ± 49.7	0.393	106.3 ± 42.8	> 0.99
ALT [U/l]	26.3 ± 12.6		18.6 ± 10.9		26.9 ± 15.2		30.4 ± 15.8		27.8 ± 17.6	
	42.2 ± 19.8	< 0.001	24.4 ± 14.5	0.197	24.5 ± 9.1	0.961	26.5 ± 6.0	0.872	25.4 ± 5.4	0.999
AST [U/l]	20.7 ± 7.2		17.6 ± 5.6		21.3 ± 8.5		20.2 ± 6.3		23.2 ± 10.6	
	31.7 ± 16.8	< 0.001	21.3 ± 9.0	0.321	20.6 ± 6.8	> 0.99	21.2 ± 7.4	0.999	20.6 ± 6.2	0.990
GGT [U/l]	42.0 ± 40.9		36.6 ± 65.0		39.3 ± 68.1		31.2 ± 31.7		18.0 ± 8.4	
	80.1 ± 91.6	0.034	51.8 ± 108.9	0.818	30.8 ± 21.0	0.991	34.3 ± 26.5	> 0.99	48.3 ± 20.7	0.918
CRP [mg/l]	15.5 ± 12.4		11.4 ± 14.1		7.4 ± 6.3		6.8 ± 6.0		4.2 ± 0.4	
	16.0 ± 21.4	> 0.99	10.4 ± 13.5	0.998	7.3 ± 5.8	> 0.99	6.4 ± 2.4	> 0.99	5.5 ± 0.8	> 0.99
HSI	63.6 ± 10.1		49.3 ± 7.8		47.9 ± 6.9		50.7 ± 7.8		46.3 ± 7.2	
	65.2 ± 9.7	0.877	51.3 ± 8.2	0.770	50.7 ± 7.3	0.602	51.3 ± 8.4	> 0.99	53.1 ± 5.9	0.523
FIB-4	0.9 ± 0.8		0.9 ± 0.6		1.0 ± 0.8		0.7 ± 0.4		0.7 ± 0.2	
	0.9 ± 0.6	0.998	0.8 ± 0.4	> 0.99	0.9 ± 0.5	0.932	0.9 ± 0.4	0.640	1.0 ± 0.5	0.910

Data are reported as mean ± SD. Follow-up data were between subgroups for each time-point. Statistical significance was assessed by two-way ANOVA with Šidák’s test for multiple comparisons. *N (%)* number of individuals, *BMI* Body Mass Index, *SG* sleeve gastrectomy, *RYGB* Roux-en-Y gastric bypass, *EWL (%)* excess weight loss, *TWL (%)* total weight loss, *Hb* hemoglobin, *HDL* high-density lipoprotein, *LDL* low-density lipoprotein, *AST* aspartate aminotransferase, *ALT* alanine aminotransferase, *GGT* gamma-glutamyl transpeptidase, *CRP* C-reactive protein, *HSI* hepatic steatosis index, *FIB-4* fibrosis-4 Index

### T2D Remission and NAFLD at Long-term Follow-up Depending on Type of Surgery

**Fig. 3:**
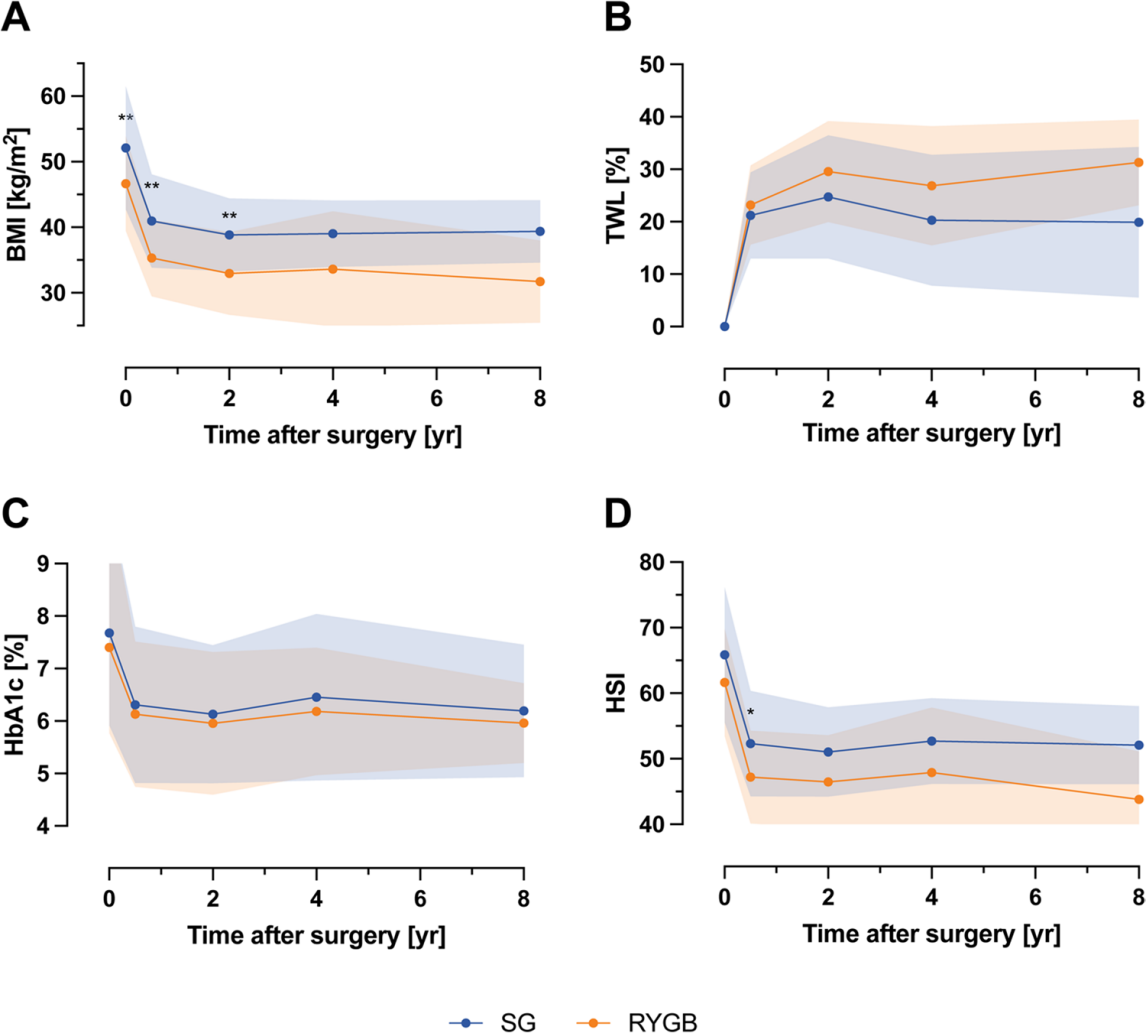
Comparison between patients with SG and RYGB. BMI (**A**), TWL (**B**), HbA1c (**C**), and HSI (**D**) during long-term follow-up. BMI, Body Mass Index; SG, sleeve gastrectomy; RYGB, Roux-en-Y gastric bypass; TWL (%), total weight loss; HSI, hepatic steatosis index; yr, years; *p < 0.05

Regarding type of surgery, there was no significant difference in T2D remission rates, total weight loss, and HbA1c at long-term follow-up between SG and RYGB. Patients who have had RYGB (*n* = 33) showed greater improvement in HSI at short-term follow-up (*p* < 0.05), but not long-term (Fig. 3, Table S2).

## Discussion

In this retrospective analysis, we aimed to understand whether the presence of NAFLD in BS patients with T2D may affect the long-term remission rates of T2D following different surgical procedures. Here, we found that the presence of low-grade hepatic steatosis at the time of surgery was positively associated with long-term remission rates of T2D up to 8 years following both, RYGB and SG. According to previous research, T2D remission rates vary between 24 and 84% depending on patient baseline characteristics and the type of surgery with slightly superior efficacy of RYGB [24]. The prevalence of NAFLD varies among subgroups of patients with T2D identified by cluster analyses and occurs in up to 77% of patients with obesity and T2D [25]. Recently, liver steatosis at the time of surgery has been found to predict a higher chance of diabetes remission 5 years following RYGB suggesting primordial importance of intrahepatic fat. The analysis was performed on patients who underwent RYGB only [16].

In our cohort, T2D remission rates were 53.8% (*n* = 28) and 50.0% (*n* = 8) at 4-year and 8-year follow-up. In patients with T2D remission, TWL% at long-term was significantly greater compared to patients without T2D remission. Remission rates were highest in steatosis grade 1, which was accompanied by lower HbA1c, triglycerides, AST, ALT, and GGT levels at baseline. NAFLD is strictly linked to peripheral and hepatic insulin resistance and associated with pancreatic β cell dysfunction, even though a comprehensive understanding of the association between β-cell dysfunction and NAFLD is still missing [26]. A decrease in liver and pancreas fat content is required for remission of T2D, which is considered to be dependent upon capacity for β cell recovery [27]. Prasad et al. prospectively assessed measures of ß-cell function and found that preintervention β cell function and its change after RYGB predicted remission, but not changes in weight loss or insulin sensitivity [28]. Short-term improvements in beta-cell function using an intravenous glucose tolerance test were similar between SG and RYGB. However, it remained unclear if longer-term outcomes are better after RYGB due to greater weight loss [29].

There are only 13 observational studies evaluating the effects of BS on histopathological NAFLD and most of them assessed the effect of RYGB [30]. In general, improvements in steatosis, inflammation and fibrosis were reported [30]. In the only randomized controlled trial available, RYGB and SG were found to be similarly effective in reducing hepatic steatosis, with an almost complete clearance of liver fat 1 year after RYGB (100%) and SG (94%) assessed by magnetic resonance imaging [31]. In our cohort, type of surgery did neither affect the chance of achieving T2D remission, nor HSI score at long-term follow-up, presuming because weight reduction has to be considered as the main driver for ß-cell recovery. SG led to similar changes in total weight loss compared with RYGB, even though SG as primary intervention is considered to be inferior to RYGB in terms of weight loss (average weight loss at 1 year 25% vs. 31% with RYGB) and diabetes remission in the short and long term [32]. To establish predictive factors of diabetes remission after BS is of great clinical interest. Even though the DiaRem-Score is considered a valid, simple, and reproducible tool to predict the outcome of T2D after BS, it might not be complete since BMI and extent of weight loss were not considered in the model [33]. In our multivariate analysis, higher BMI (OR 1.19; *p* = 0.002), lower preoperative insulin use (OR 0.8; *p* = 0.001), and lower HbA1c (OR 0.29; *p* < 0.001) at baseline were associated with long-term T2D remission (*n* = 16) (Table S4), which is in line with previous data [16]. Our results support the hypothesis that predictors for diabetes remission after BS may be classified as belonging to either indirect indices for preserved pancreatic beta-cell function, including younger age, shorter duration of diabetes, higher C-peptide, lower HbA1c and no use of insulin or reflect the potential for an insulin resistance reduction, including higher baseline BMI and visceral fat area [34]. In our cohort, preserved beta-cell function seemed to be the major determinant of remission which links to the concept of metabolic memory, since patients with poor glycemic control and insulin use at baseline were more likely to experience treatment failure with regard to diabetes remission despite relevant weight loss.

Since it was not feasible to perform liver biopsy 8 years post surgical intervention, we calculated HSI as simple and efficient screening tool for NAFLD at long-term follow-up [35]. We observed a significant improvement in HSI over time in patients without and with T2D remission, but HSI at long-term follow-up was not significantly different between the groups and still indicating a high likelihood of NAFLD diagnosis. Even though diagnostic accuracy of HSI might be limited in the postbariatric population, this aspect may further support the results of Vangoitsenhoven et al. that the preoperative presence of steatosis plays a weight-independent additive predictive role, but is of secondary importance compared to indirect markers of ß-cell reserve and weight loss [16]. Prospective studies are needed to further evaluate whether the degree of hepatic steatosis might serve as a surrogate parameter for glycemic control predicting T2D remission over time following BS T2D remission over time. The duration of the benefit of surgery in terms of diabetes remission is still unclear. In our cohort, HbA1c was still decreasing by trend at 8 years follow-up without reaching statistical significance and paralleled the decline in HSI during follow-up.

Our study has a few limitations. Here, we report a retrospective analysis, at 96-month follow-up, only 16 patients have been included into the analysis. Measurement of direct markers of ß cell function and insulin resistance were not performed.

In conclusion, the presence of NAFLD should routinely be assessed in patients with obesity and T2D and impact treatment decisions. Metabolic surgery, independent of type of surgery, should be considered at an early NAFLD stage in patients with T2D. Moreover, mechanistic studies that further elucidate the bidirectional relationship between T2D remission and NAFLD are needed.

## Supplementary Information

Below is the link to the electronic supplementary material.

Supplementary file1 (PNG 237 KB)

Supplementary file2 (DOCX 17 KB)

Supplementary file3 (DOCX 18 KB)

Supplementary file4 (DOCX 13 KB)

## Data Availability

The data that support the findings of the study are available from the corresponding author, AL, upon reasonable request.

## References

[1] Younossi ZM, Golabi P, de Avila L, Paik JM, Srishord M, Fukui N (2019). The global epidemiology of NAFLD and NASH in patients with type 2 diabetes: a systematic review and meta-analysis. J Hepatol..

[2] Targher G, Corey KE, Byrne CD, Roden M (2021). The complex link between NAFLD and type 2 diabetes mellitus - mechanisms and treatments. Nat Rev Gastroenterol Hepatol..

[3] Stefan N, Cusi K (2022). A global view of the interplay between non-alcoholic fatty liver disease and diabetes. Lancet Diabetes Endocrinol..

[4] Gastaldelli A, Cusi K (2019). From NASH to diabetes and from diabetes to NASH: Mechanisms and treatment options. JHEP Reports Elsevier.

[5] Sasaki A, Nitta H, Otsuka K, Umemura A, Baba S, Obuchi T, et al. Bariatric surgery and non-alcoholic Fatty liver disease: current and potential future treatments. Front Endocrinol (Lausanne). 2014;5. 10.3389/fendo.2014.00164 PMC4209858 25386164

[6] Lassailly G, Caiazzo R, Buob D, Pigeyre M, Verkindt H, Labreuche J (2015). Bariatric surgery reduces features of nonalcoholic steatohepatitis in morbidly obese patients. Gastroenterology.

[7] Laursen TL, Hagemann CA, Wei C, Kazankov K, Thomsen KL, Knop FK (2019). Bariatric surgery in patients with non-alcoholic fatty liver disease - from pathophysiology to clinical effects. World J Hepatol.

[8] Goldoni MB, Fontes PRO, Guimarães MM, Diedrich-Neto JA, Nogueira T, Teixeira UF, et al. Bypass vs. sleeve and its effects in non-alcoholic fatty liver disease: what is the best technique? Arq Bras Cir Dig. 2021;33:1–5. 10.1590/0102-672020200003e1549 PMC7812689 33470379

[9] Pedersen JS, Rygg MO, Serizawa RR, Kristiansen VB, Wewer Albrechtsen NJ, Gluud LL, et al. Effects of Roux-en-Y gastric bypass and sleeve gastrectomy on non-alcoholic fatty liver disease: a 12-month follow-up study with paired liver biopsies. J Clin Med. 2021;10. 10.3390/jcm10173783 PMC8432029 34501231

[10] Liu N, Funk LM. Bariatric surgery and diabetes treatment-finding the sweet spot. JAMA Surg. 2020;155. 10.1001/jamasurg.2020.0088 PMC7483969 32129803

[11] Lee Y, Doumouras AG, Yu J, Aditya I, Gmora S, Anvari M (2021). Laparoscopic sleeve gastrectomy versus laparoscopic Roux-en-Y gastric bypass: a systematic review and meta-analysis of weight loss, comorbidities, and biochemical outcomes from randomized controlled trials. Ann Surg..

[12] Sharples AJ, Mahawar K (2020). Systematic review and meta-analysis of randomised controlled trials comparing long-term outcomes of Roux-En-Y gastric bypass and sleeve gastrectomy. Obes Surg.

[13] McTigue KM, Wellman R, Nauman E, Anau J, Coley RY, Odor A, et al. Comparing the 5-year diabetes outcomes of sleeve gastrectomy and gastric bypass. JAMA Surg [Internet]. Am Med Assoc. 2020;155:e200087. 10.1001/jamasurg.2020.0087 PMC7057171 32129809

[14] Sattar N, Gill JMR. Type 2 diabetes as a disease of ectopic fat? BMC Med. 2014;12. 10.1186/s12916-014-0123-4 PMC4143560 25159817

[15] Lim EL, Hollingsworth KG, Aribisala BS, Chen MJ, Mathers JC, Taylor R (2011). Reversal of type 2 diabetes: normalisation of beta cell function in association with decreased pancreas and liver triacylglycerol. Diabetologia..

[16] Vangoitsenhoven R, Wilson RL, Cherla DV, Tu C, Kashyap SR, Cummings DE (2021). Presence of liver steatosis is associated with greater diabetes remission after gastric bypass surgery. Diabetes Care..

[17] S3-Leitlinie: Chirurgie der Adipositas und metabolischer Erkrankungen. 2018; Available from: https://www.awmf.org/uploads/tx_szleitlinien/088-001l_S3_Chirurgie-Adipositas-metabolische-Erkrankugen_2018-02.pdf. Accessed 28 Apr 2022.

[18] Kleiner DE, Brunt EM, Van Natta M, Behling C, Contos MJ, Cummings OW (2005). Design and validation of a histological scoring system for nonalcoholic fatty liver disease. Hepatology [Internet]. Hepatology.

[19] Brethauer SA, Kim J, El Chaar M, Papasavas P, Eisenberg D, Rogers A (2015). Standardized outcomes reporting in metabolic and bariatric surgery. Surg Obes Relat Dis..

[20] Riddle MC, Cefalu WT, Evans PH, Gerstein HC, Nauck MA, Oh WK (2021). Consensus Report: definition and interpretation of remission in type 2 diabetes. Diabetes Care..

[21] Fedchuk L, Nascimbeni F, Pais R, Charlotte F, Housset C, Ratziu V (2014). Performance and limitations of steatosis biomarkers in patients with nonalcoholic fatty liver disease. Aliment Pharmacol Ther..

[22] Sterling RK, Lissen E, Clumeck N, Sola R, Correa MC, Montaner J (2006). Development of a simple noninvasive index to predict significant fibrosis in patients with HIV/HCV coinfection. Hepatology..

[23] Angulo P, Hui JM, Marchesini G, Bugianesi E, George J, Farrell GC (2007). The NAFLD fibrosis score: a noninvasive system that identifies liver fibrosis in patients with NAFLD. Hepatology..

[24] Park JY (2018). Prediction of type 2 diabetes remission after bariatric or metabolic surgery. J Obes Metab Syndr..

[25] Stefan N, Cusi K (2022). A global view of the interplay between non-alcoholic fatty liver disease and diabetes. Lancet Diabetes Endocrinol..

[26] Chen X, Xiao J, Pang J, Chen S, Wang Q, Ling W. Pancreatic β-Cell dysfunction is associated with nonalcoholic fatty liver disease. Nutrients [Internet]. Multidisciplinary Digital Publishing Institute (MDPI); 2021;13. 10.3390/nu13093139 PMC8468093 34579016

[27] Taylor R, Al-Mrabeh A, Zhyzhneuskaya S, Peters C, Barnes AC, Aribisala BS (2018). Remission of human type 2 diabetes requires decrease in liver and pancreas fat content but is dependent upon capacity for β cell recovery. Cell Metab Cell Press.

[28] Prasad M, Mark V, Ligon C, Dutia R, Nair N, Shah A, et al. Role of the gut in the temporal changes of β-cell function after gastric bypass in individuals with and without diabetes remission. Diabetes Care [Internet]. Am Diab Assoc. 2022;45:469–76. 10.2337/dc21-1270 PMC8914419 34857533

[29] Mullally JA, Febres GJ, Bessler M, Korner J. Sleeve gastrectomy and Roux-en-Y gastric bypass achieve similar early improvements in beta-cell function in obese patients with type 2 diabetes. Sci Reports. 2019;9:1–7. 10.1038/s41598-018-38283-y PMC6372630 30755673

[30] Laursen TL, Hagemann CA, Wei C, Kazankov K, Thomsen KL, Knop FK (2019). Bariatric surgery in patients with non-alcoholic fatty liver disease - from pathophysiology to clinical effects. World J Hepatol..

[31] Seeberg KA, Borgeraas H, Hofsø D, Småstuen MC, Kvan NP, Grimnes JO (2022). Gastric bypass versus sleeve gastrectomy in type 2 diabetes: effects on hepatic steatosis and fibrosis : a randomized controlled trial. Ann Intern Med.

[32] McTigue KM, Wellman R, Nauman E, Anau J, Coley RY, Odor A, et al. Comparing the 5-year diabetes outcomes of sleeve gastrectomy and gastric bypass. JAMA Surg. 2020;155:e200087. 10.1001/jamasurg.2020.0087 PMC7057171 32129809

[33] Aminian A, Brethauer SA, Kashyap SR, Kirwan JP, Schauer PR (2014). DiaRem score: external validation. Lancet Diabetes Endocrinol.

[34] Park JY. Prediction of type 2 diabetes remission after bariatric or metabolic surgery. J Obes Metab Syndr. 2018;27:213. 10.7570/jomes.2018.27.4.213 PMC6513303 31089566

[35] Lee JH, Kim D, Kim HJ, Lee CH, Yang JI, Kim W, et al. Hepatic steatosis index: a simple screening tool reflecting nonalcoholic fatty liver disease. Dig Liver Dis. 42:503–8. 10.1016/j.dld.2009.08.002 19766548

