# Supplementary figures and images for: Low-Grade Hepatic Steatosis Is Associated with Long-term Remission of Type 2 Diabetes Independent of Type of Bariatric-Metabolic Surgery
Source: Obes Surg. 2022 Dec 12;33(2):530–8. doi: 10.1007/s11695-022-06406-0 (PMC9889466; doi:10.1007/s11695-022-06406-0)

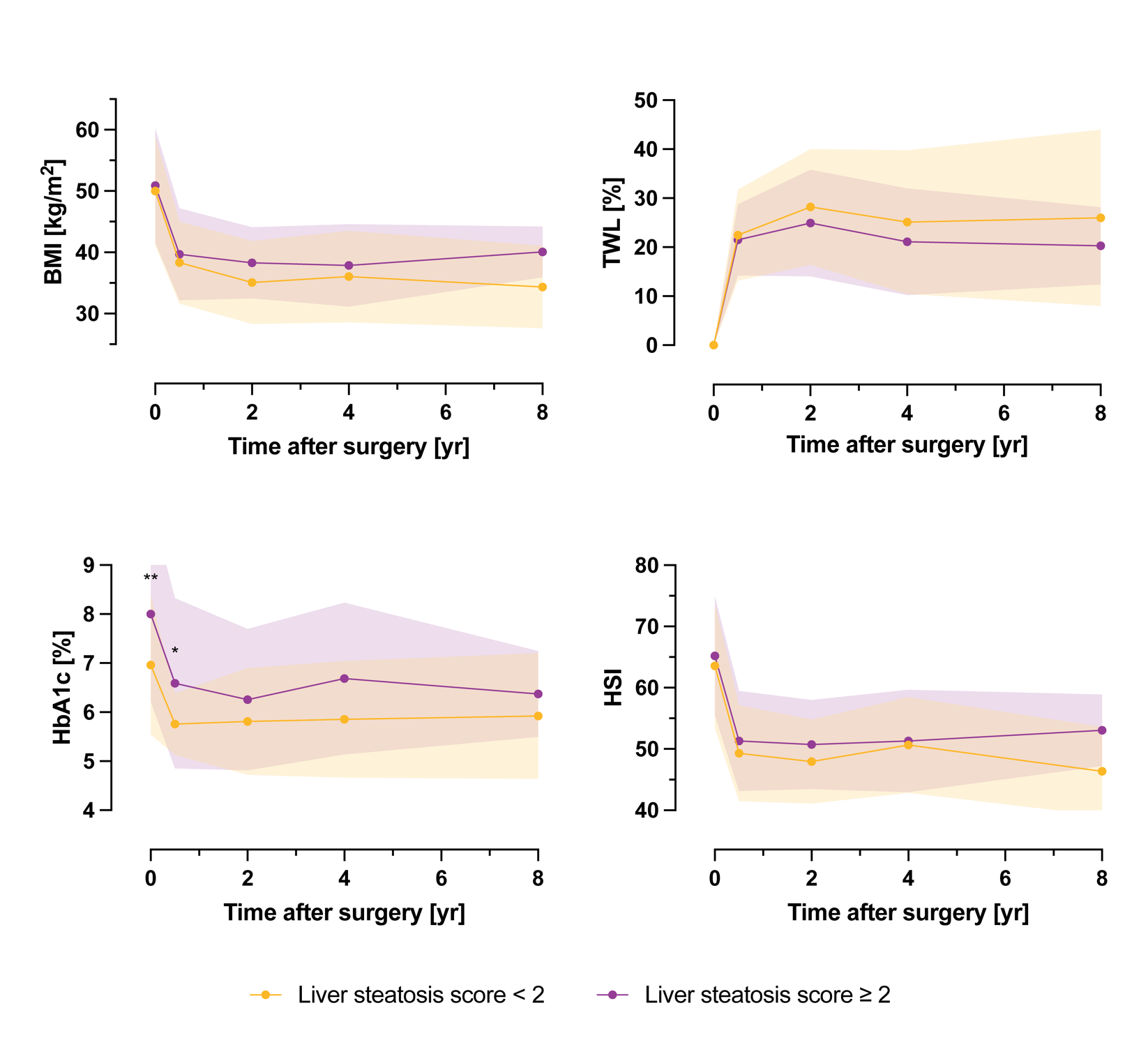

Supplement: Supplementary file 1 — Supplementary file1 (PNG 237 KB) [file 11695_2022_6406_MOESM1_ESM.png]
