# Supplementary material for: Low-Grade Hepatic Steatosis Is Associated with Long-term Remission of Type 2 Diabetes Independent of Type of Bariatric-Metabolic Surgery
Source: Obes Surg. 2022 Dec 12;33(2):530–8. doi: 10.1007/s11695-022-06406-0 (PMC9889466; doi:10.1007/s11695-022-06406-0)
Supplement: Supplementary file 2 — Supplementary file2 (DOCX 17 KB) [file 11695_2022_6406_MOESM2_ESM.docx]

**Table S1.** Comparison between T2D patients without remission (Top) and with remission (Bottom).

| **Parameter** | **Baseline** | **p** | **6M-FU** | **p** | **24M-FU** | **p** | **48M-FU** | **p** | **96M-FU** | **p** |
| --- | --- | --- | --- | --- | --- | --- | --- | --- | --- | --- |
| **no T2D Remission [n]** | 47 |  | 43 |  | 30 |  | 24 |  | 8 |  |
| **T2D Remission [n]** | 60 |  | 57 |  | 44 |  | 28 |  | 8 |  |
| **BMI [kg/m^2^]** | 48.4 ± 8.8 |  | 38.4 ± 7.0 |  | 36.8 ± 6.5 |  | 37.5 ± 6.4 |  | 38.5 ± 5.2 |  |
|  | 52.0 ± 8.9 | 0.081 | 39.6 ± 7.3 | 0.954 | 37.0 ± 6.3 | >0.99 | 36.8 ± 7.5 | >0.99 | 35.4 ± 7.3 | >0.99 |
| **EWL [%]** |  |  | 43.1 ± 16.3 |  | 46.8 ± 25.6 |  | 41.0 ± 30.3 |  | 31.7 ± 25.9 |  |
|  |  |  | 46.9 ± 16.4 | 0.884 | 57.8 ± 23.2 | 0.177 | 54.1 ± 30.2 | 0.184 | 66.1 ± 24.8 | 0.023 |
| **TWL [%]** |  |  | 20.0 ± 8.0 |  | 21.5 ± 11.0 |  | 18.9 ± 11.8 |  | 15.5 ± 11.3 |  |
|  |  |  | 23.4 ± 7.8 | 0.320 | 29.8 ± 10.3 | 0.003 | 26.0 ± 12.2 | 0.057 | 33.3 ± 9.5 | 0.004 |
| **HbA1c [%]** | 8.7 ± 1.4 |  | 7.3 ± 1.5 |  | 7.3 ± 1.2 |  | 7.4 ± 1.4 |  | 6.9 ± 0.9 |  |
|  | 6.6 ± 1.2 | <0.001 | 5.4 ± 0.5 | <0.001 | 5.2 ± 0.4 | <0.001 | 5.4 ± 0.5 | <0.001 | 5.2 ± 0.4 | 0.014 |
| **Hb [g/dl]** | 13.9 ± 1.4 |  | 13.7 ± 1.1 |  | 13.3 ± 1.1 |  | 13.7 ± 1.5 |  | 12.8 ± 2.1 |  |
|  | 13.9 ± 1.5 | >0.99 | 13.4 ± 1.4 | 0.892 | 13.3 ± 1.5 | >0.99 | 13.5 ± 1.3 | 0.994 | 13.3 ± 1.0 | 0.943 |
| **Leukocytes [10^9^/l]** | 9.4 ± 2.7 |  | 9.0 ± 2.6 |  | 8.6 ± 2.1 |  | 8.7 ± 2.0 |  | 7.6 ± 1.4 |  |
|  | 9.8 ± 2.3 | 0.922 | 8.7 ± 2.0 | 0.980 | 7.4 ± 2.4 | 0.142 | 7.9 ± 2.2 | 0.692 | 7.6 ± 2.4 | >0.99 |
| **Platelet Count [10^9^/l]** | 288.4 ± 77.5 |  | 284.8 ± 75.1 |  | 290.9 ± 79.3 |  | 295.1 ± 69.4 |  | 263.0 ± 63.8 |  |
|  | 296.1 ± 75.9 | 0.990 | 286.4 ± 76.4 | >0.99 | 266.4 ± 81.5 | 0.607 | 272.3 ± 67.1 | 0.803 | 265.8 ± 46.8 | >0.99 |
| **Triglycerides [mg/dl]** | 275.9 ± 173.7 |  | 217.8 ± 100.5 |  | 249.2 ± 131.0 |  | 312.2 ± 198.8 |  | 243.7 ± 195.5 |  |
|  | 211.0 ± 104.2 | 0.031 | 169.8 ± 80.9 | 0.222 | 153.1 ± 81.7 | 0.004 | 153.5 ± 68.0 | <0.001 | 140.0 ± 57.8 | 0.361 |
| **HDL [mg/dl]** | 40.4 ± 13.5 |  | 43.3 ± 12.7 |  | 47.8 ± 13.4 |  | 51.0 ± 17.2 |  | 58.2 ± 18.4 |  |
|  | 40.9 ± 13.4 | >0.99 | 42.9 ± 9.8 | >0.99 | 57.3 ± 14.8 | 0.016 | 63.5 ± 13.7 | 0.006 | 64.8 ± 9.7 | 0.877 |
| **LDL [mg/dl]** | 100.2 ± 46.4 |  | 99.5 ± 42.6 |  | 103.3 ± 37.2 |  | 103.7 ± 56.7 |  | 111.8 ± 39.3 |  |
|  | 99.8 ± 35.4 | >0.99 | 106.1 ± 36.4 | 0.937 | 94.9 ± 41.9 | 0.917 | 85.9 ± 29.2 | 0.533 | 100.7 ± 38.8 | 0.989 |
| **ALT [U/l]** | 37.2 ± 20.2 |  | 24.4 ± 15.7 |  | 25.5 ± 12.8 |  | 28.3 ± 6.2 |  | 23.6 ± 4.4 |  |
|  | 35.1 ± 18.0 | 0.958 | 20.2 ± 11.2 | 0.609 | 25.4 ± 11.3 | >0.999 | 27.7 ± 13.8 | >0.999 | 29.8 ± 18.4 | 0.922 |
| **ALT [U/l]** | 37.2 ± 20.2 |  | 24.4 ± 15.7 |  | 25.5 ± 12.8 |  | 28.3 ± 6.2 |  | 23.6 ± 4.4 |  |
|  | 35.1 ± 18.0 | 0.966 | 20.2 ± 11.2 | 0.935 | 25.4 ± 11.3 | >0.99 | 27.7 ± 13.8 | >0.99 | 29.8 ± 18.4 | 0.651 |
| **GGT [U/l]** | 74.3 ± 104.2 |  | 59.0 ± 127.5 |  | 32.4 ± 31.6 |  | 35.1 ± 29.9 |  | 40.5 ± 23.7 |  |
|  | 58.1 ± 48.0 | 0.753 | 35.6 ± 55.5 | 0.419 | 35.4 ± 54.0 | >0.99 | 31.2 ± 27.3 | >0.99 | 22.0 ± 14.2 | 0.990 |
| **CRP [mg/l]** | 17.3 ± 24.5 |  | 10.1 ± 13.0 |  | 7.6 ± 5.4 |  | 6.7 ± 2.5 |  | 5.1 ± 1.1 |  |
|  | 14.6 ± 11.2 | 0.825 | 11.3 ± 14.2 | 0.996 | 7.2 ± 6.3 | >0.99 | 6.3 ± 5.2 | >0.99 | 4.3 ± 0.5 | >0.99 |
| **HSI** | 62.3 ± 9.7 |  | 50.6 ± 8.0 |  | 49.6 ± 7.4 |  | 51.7 ± 8.1 |  | 51.9 ± 6.3 |  |
|  | 66.3 ± 9.6 | 0.087 | 50.4 ± 8.1 | >0.99 | 49.5 ± 7.1 | >0.99 | 50.4 ± 8.1 | 0.987 | 46.7 ± 7.7 | 0.766 |
| **FIB-4** | 0.9 ± 0.6 |  | 0.9 ± 0.4 |  | 0.9 ± 0.5 |  | 0.8 ± 0.3 |  | 0.8 ± 0.5 |  |
|  | 0.8 ± 0.6 | 0.739 | 0.8 ± 0.5 | 0.960 | 0.8 ± 0.6 | >0.99 | 0.7 ± 0.3 | >0.99 | 0.7 ± 0.1 | 0.999 |

Data are reported as mean ± SD. Follow-up data were between subgroups for each time-point. Statistical significance was assessed by two-way ANOVA with Šidák’s test for multiple comparisons. N (%), number of individuals; T2D, Type 2 Diabetes; BMI, Body Mass Index; EWL, excess weight loss; TWL, total weight loss; Hb, hemoglobin; HDL, high-density lipoprotein; LDL, low-density lipoprotein;. ALT, alanine aminotransferase; AST, aspartate aminotransferase; GGT, gamma-glutamyl transpeptidase; CRP, C-reactive protein; HSI, hepatic steatosis index; FIB-4, Fibrosis-4 Index.
