# Supplementary material for: Low-Grade Hepatic Steatosis Is Associated with Long-term Remission of Type 2 Diabetes Independent of Type of Bariatric-Metabolic Surgery
Source: Obes Surg. 2022 Dec 12;33(2):530–8. doi: 10.1007/s11695-022-06406-0 (PMC9889466; doi:10.1007/s11695-022-06406-0)
Supplement: Supplementary file 3 — Supplementary file3 (DOCX 18 KB) [file 11695_2022_6406_MOESM3_ESM.docx]

**Table S2.** Comparison between patients with SG (Top) and RYGB (Bottom).

| **Parameter** | **Baseline** | **p** | **6M-FU** | **p** | **24M-FU** | **p** | **48M-FU** | **p** | **96M-FU** | **p** |
| --- | --- | --- | --- | --- | --- | --- | --- | --- | --- | --- |
| **SG [n]** | 74 |  | 68 |  | 51 |  | 35 |  | 11 |  |
| **RYGB [n]** | 33 |  | 32 |  | 23 |  | 17 |  | 5 |  |
| **T2D Remission [n (%)]** | 40 (54.1) |  | 38 (55.9) |  | 29 (56.9) |  | 17 (48.6) |  | 4 (36.4) |  |
|  | 20 (60.6) | 0.528 | 19 (59.4) | 0.742 | 15 (65.2) | 0.498 | 11 (64.7) | 0.274 | 4 (80.0) | 0.107 |
| **BMI [kg/m^2^]** | 52.1 ± 9.4 |  | 41.0 ± 7.1 |  | 38.8 ± 5.6 |  | 39 ± 5.1 |  | 39.4 ± 4.8 |  |
|  | 46.6 ± 7.2 | 0.002 | 35.3 ± 5.9 | 0.002 | 33.0 ± 6.3 | 0.007 | 33.6 ± 8.8 | 0.062 | 31.7 ± 6.3 | 0.315 |
| **EWL [%]** |  |  | 41.9 ± 14.5 |  | 47.4 ± 21.7 |  | 39.3 ± 25.6 |  | 36.5 ± 25.8 |  |
|  |  |  | 52.5 ± 18.2 | 0.106 | 65.8 ± 26.9 | 0.005 | 64.2 ± 33.9 | 0.001 | 71.6 ± 27.9 | 0.029 |
| **TWL [%]** |  |  | 21.2 ± 8.3 |  | 24.7 ± 11.7 |  | 20.3 ± 12.5 |  | 19.9 ± 14.4 |  |
|  |  |  | 23.2 ± 7.5 | 0.953 | 29.6 ± 9.6 | 0.246 | 26.9 ± 11.4 | 0.145 | 31.3 ± 8.2 | 0.226 |
| **HbA1c [%]** | 7.7 ± 1.8 |  | 6.3 ± 1.5 |  | 6.1 ± 1.3 |  | 6.5 ± 1.6 |  | 6.2 ± 1.3 |  |
|  | 7.4 ± 1.6 | 0.908 | 6.1 ± 1.4 | 0.988 | 6.0 ± 1.4 | 0.994 | 6.2 ± 1.2 | 0.980 | 6.0 ± 0.8 | >0.99 |
| **Hb [g/dl]** | 14.0 ± 1.7 |  | 13.8 ± 1.4 |  | 13.6 ± 1.4 |  | 13.9 ± 1.5 |  | 13.1 ± 2.0 |  |
|  | 13.9 ± 1.2 | 0.997 | 13.3 ± 1.2 | 0.435 | 12.7 ± 1.2 | 0.052 | 13.2 ± 1.3 | 0.374 | 13.1 ± 0.5 | >0.99 |
| **Leukocytes [10^9^/l]** | 9.7 ± 2.6 |  | 9.3 ± 2.4 |  | 8.0 ± 2.3 |  | 8.5 ± 2.2 |  | 7.8 ± 1.2 |  |
|  | 9.5 ± 2.3 | 0.988 | 8.1 ± 1.9 | 0.082 | 7.9 ± 2.7 | >0.99 | 8.1 ± 2.3 | 0.990 | 7.3 ± 3.2 | 0.998 |
| **Platelet Count [10^9^/l]** | 282.7 ± 76.2 |  | 281.0 ± 79.7 |  | 270.1 ± 85.0 |  | 279.2 ± 69.4 |  | 268.8 ± 50.9 |  |
|  | 315.4 ± 73.1 | 0.117 | 293.9 ± 66.2 | 0.939 | 290.2 ± 71.1 | 0.817 | 290.4 ± 68.2 | 0.991 | 254.8 ± 66.0 | 0.999 |
| **Triglycerides [mg/dl]** | 257.2 ± 151.2 |  | 209.2 ± 97.1 |  | 204.5 ± 111.7 |  | 255.2 ± 174.5 |  | 223.7 ± 172.4 |  |
|  | 199.4 ± 110.2 | 0.134 | 150.8 ± 67.6 | 0.142 | 165.0 ± 118.3 | 0.705 | 170.9 ± 124.9 | 0.118 | 121.8 ± 18.2 | 0.507 |
| **HDL [mg/dl]** | 39.4 ± 13.9 |  | 41.9 ± 11.1 |  | 52.2 ± 15.7 |  | 55.9 ± 17.3 |  | 55.7 ± 14.9 |  |
|  | 43.8 ± 12.0 | 0.478 | 45.7 ± 10.9 | 0.656 | 56.3 ± 13.0 | 0.739 | 61.6 ± 14.7 | 0.582 | 72.6 ± 6.5 | 0.113 |
| **LDL [mg/dl]** | 94.6 ± 39.8 |  | 106.4 ± 38.5 |  | 105.6 ± 40.3 |  | 96.0 ± 45.6 |  | 105.6 ± 37.2 |  |
|  | 110.9 ± 39.1 | 0.247 | 96.3 ± 40.6 | 0.742 | 82.3 ± 35.3 | 0.113 | 88.7 ± 39.4 | 0.981 | 108.8 ± 44.4 | >0.99 |
| **ALT [U/l]** | 34.8 ± 16.9 |  | 22.4 ± 14.5 |  | 25.1 ± 10.7 |  | 27.6 ± 12.1 |  | 28.4 ± 15.5 |  |
|  | 38.9 ± 23.1 | 0.667 | 21.3 ± 11.0 | 0.999 | 26.4 ± 14.4 | 0.998 | 28.9 ± 8.2 | >0.99 | 23.2 ± 6.9 | 0.974 |
| **AST [U/l]** | 27.5 ± 15.1 |  | 20.4 ± 9.0 |  | 21.3 ± 7.8 |  | 20.8 ± 7.8 |  | 22.5 ± 10.5 |  |
|  | 27.2 ± 14.6 | >0.99 | 18.7 ± 5.4 | 0.952 | 20.0 ± 7.0 | 0.991 | 20.8 ± 4.9 | >0.99 | 21.2 ± 3.6 | >0.99 |
| **GGT [U/l]** | 64.2 ± 62.5 |  | 54.2 ± 109.6 |  | 38.2 ± 52.7 |  | 34.2 ± 26.2 |  | 35.9 ± 24.0 |  |
|  | 67.8 ± 106.9 | >0.99 | 27.6 ± 40.2 | 0.344 | 25.3 ± 24.6 | 0.958 | 30.8 ± 33.2 | >0.99 | 21.0 ± 7.7 | 0.997 |
| **CRP [mg/l]** | 15.4 ± 12.2 |  | 12.6 ± 16.1 |  | 8.4 ± 7.0 |  | 6.8 ± 4.9 |  | 5.0 ± 0.9 |  |
|  | 16.8 ± 27.8 | 0.991 | 7.0 ± 4.0 | 0.202 | 5.1 ± 0.6 | 0.851 | 5.9 ± 1.9 | >0.99 | 4.2 ± 0.4 | >0.99 |
| **HSI** | 65.9 ± 10.3 |  | 52.3 ± 8.1 |  | 51.0 ± 6.8 |  | 52.7 ± 6.5 |  | 52.1 ± 6.0 |  |
|  | 61.6 ± 8.2 | 0.77 | 47.2 ± 7.1 | 0.022 | 46.5 ± 7.1 | 0.138 | 47.9 ± 9.9 | 0.239 | 43.8 ± 7.3 | 0.375 |
| **FIB-4** | 1.0 ± 0.7 |  | 0.9 ± 0.6 |  | 1.0 ± 0.7 |  | 0.8 ± 0.4 |  | 0.8 ± 0.4 |  |
|  | 0.7 ± 0.5 | 0.275 | 0.7 ± 0.3 | 0.473 | 0.7 ± 0.3 | 0.249 | 0.7 ± 0.3 | 0.979 | 0.8 ± 0.2 | >0.99 |

Data are reported as mean ± SD. Follow-up data were between subgroups for each time-point. Statistical significance was assessed by two-way ANOVA with Šidák’s test for multiple comparisons for continuous variables and Chi-square test for categorical variables. N (%), number of individuals; BMI, Body Mass Index; SG, sleeve gastrectomy; RYGB, Roux-en-Y gastric bypass; EWL (%), excess weight loss; TWL (%), total weight loss; Hb, hemoglobin. HDL, high-density lipoprotein. LDL, low-density lipoprotein. ALT, alanine aminotransferase. AST, aspartate aminotransferase. GGT gamma-glutamyl transpeptidase. CRP, C-reactive protein. HIS, hepatic steatosis index; FIB-4, Fibrosis-4 Index.
