# Supplementary material for: Low-Grade Hepatic Steatosis Is Associated with Long-term Remission of Type 2 Diabetes Independent of Type of Bariatric-Metabolic Surgery
Source: Obes Surg. 2022 Dec 12;33(2):530–8. doi: 10.1007/s11695-022-06406-0 (PMC9889466; doi:10.1007/s11695-022-06406-0)
Supplement: Supplementary file 4 — Supplementary file4 (DOCX 13 KB) [file 11695_2022_6406_MOESM4_ESM.docx]

**Table S3** Multiple logistic regression analysis between long-term T2D remission outcome and baseline variables.

| **Baseline variables** | **OR** | **95% CI** | **P value** | **Z value** |
| --- | --- | --- | --- | --- |
| **Sex [f]** | 1.01 | 0.26-4.12 | 0.985 | 0.02 |
| **Age** | 1.04 | 0.98-1.12 | 0.218 | 1.23 |
| **BMI** | 1.19 | 1.079-1.34 | 0.002 | 3.14 |
| **HbA1c** | 0.29 | 0.15-0.50 | <0.001 | 3.97 |
| **Preoperative insulin use** | 0.08 | 0.01-0.35 | 0.001 | 3.18 |
| **Preoperative oral antidiabetic agents** | 0.39 | 0.09-1.48 | 0.186 | 1.32 |
| **Liver steatosis** | 1.42 | 0.36-5.92 | 0.619 | 0.50 |
| **Liver inflammation** | 0.40 | 0.07-2.04 | 0.281 | 1.08 |
| **Hepatocyte ballooning** | 0.38 | 0.07-1.87 | 0.250 | 1.15 |
| **Liver fibrosis** | 0.48 | 0.12-1.80 | 0.282 | 1.08 |

Odds ratio (OR) with 95% confidence intervals (CI) and p values. Statistical significance was assessed using a multiple logistic regression model with main effects for each variable. BMI, Body Mass Index.
